# An appetite for understanding appetite

**DOI:** 10.1371/journal.pbio.2002838

**Published:** 2017-05-31

**Authors:** Gabriel Gasque

**Affiliations:** Public Library of Science, San Francisco, California, United States of America

I have a sweet tooth, and I will always choose any pastry over spinach salad. I also know that many readers will share this preference. However, I am also aware that an unbalanced diet, in quantity and quality, is associated with serious health problems, including type 2 diabetes, metabolic syndrome, cardiovascular diseases, and cancer. It has therefore become a pressing question in basic and translational neurobiology to understand both how animals choose what to eat and in what quantities.

The feelings of hunger and satiety are thought to arise from crosstalk between the digestive system, the energy-storing cells (such as adipocytes), and ultimately, the brain.

In an effort to understand the mechanisms that drive hunger, trigger satiety, and modulate appetite, scientists often turn to invertebrate model organisms. Invertebrates are easy to raise and to manipulate experimentally. Their reduced complexity allows a finer and quicker dissection of the cellular and molecular pathways controlling appetite. For example, while the human brain has 86 billion neurons, the brain of the vinegar fly *Drosophila melanogaster* contains about 250,000, and the nervous system of the nematode *Caenorhabditis elegans* is composed of only 302 neurons. Scale aside, the neurons, gut, and other tissues in these simpler organisms are very similar in form, function, and genetics to those of humans.

In all animals, regardless of their size and complexity, health is promoted by the ingestion of appropriate quantities of specific nutrients, and these quantities are dictated by the internal nutritional state: too little or too much of a specific nutrient can be detrimental. Therefore, animals must execute precise control over the consumption of these nutrients. *D*. *melanogaster* obtains its proteins from the yeast that grows in rotting fruit, and yeast deprivation induces in the fly both a specific appetite for proteins and a reduction in the capacity to lay eggs. A recent study published in *PLOS Biology* investigated the factors that control this homeostatic appetite for proteins [1], finding that both dietary essential amino acids (those that the animals cannot synthetize themselves and so need to be obtained from food) and the bacteria that live in the gut of flies are key modulators of the appetite for proteins.

The authors first observed that the behavioral and physiological effects of a diet poor in protein could be reproduced by removing from the diet only the essential amino acids. Furthermore, if the authors transformed a non-essential amino acid into an essential amino acid (by genetically disrupting the flies’ ability to make it), its removal from the diet induced the same increase in appetite for proteinaceous food as the removal of any of the original essential amino acids.

Intriguingly, however, flies with an appropriate gut microbiome did not develop this appetite for proteins when fed a diet low in amino acids, and only partially reduced the number of eggs they laid. Specifically, two gut bacteria species, *Acetobacter pomorum* and *Lactobacillus sp*., when introduced with an otherwise amino acid-poor diet, suppressed the physiological and behavioral changes. However, neither bacterium could do it alone, which made the authors conclude that they must be working together.

**Fig 1:**
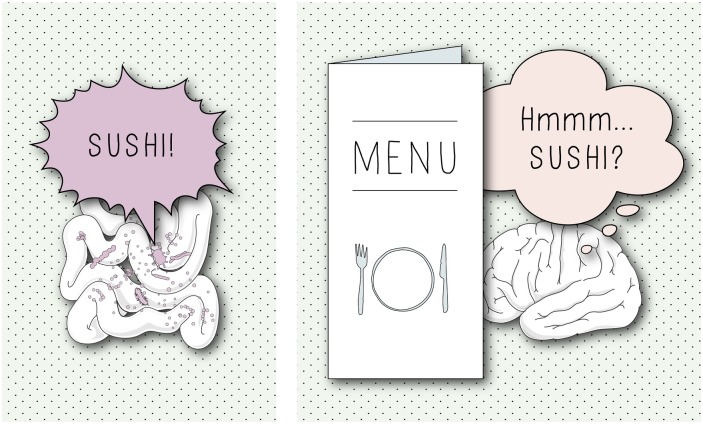
Gut bacteria can influence feeding choices. This fanciful anthropomorphic image reflects the real interaction seen between flies, their gut microbiome, and their preference for a proteinaceous diet. *Image credit*: *Gil Costa with elements from Servier Medical Art*.

While the mechanism by which these commensal bacteria rescue the behavioral and physiological responses triggered by a diet lacking essential amino acids remains to be determined, the authors did demonstrate that the bacteria were not changing the levels of amino acids within the flies. The bacteria must therefore be either promoting the better use of remaining amino acids or bypassing the lack of essential amino acids by directly activating the pathways that are sensitive to this dietary deficiency (Fig 1).

In addition to a dietary deficit in essential amino acids, another event that increases the appetite for proteins in flies is mating: mated flies eat more protein than virgin flies. A recent study published in *PLOS ONE* [2] investigated how this homeostatic appetite for protein in mated female flies is integrated with the circadian clock. The authors found that mated—but not virgin—female flies ate more amino acids at night-time; this preference for amino acids in the dark was abolished if the internal clock was disrupted by a mutation. But how can mating control appetite? The authors present evidence that a short protein called “sex peptide,” transmitted from males to females via seminal fluid, may mediate the nocturnal preference for amino acids. This makes sense, as extra supplies of amino acids will be needed to provision the fertilized eggs.

The circadian clock regulates not only feeding, but also sleep, and a paper published in *PLOS Genetics* [3] reports a study that investigated how these two activities are interconnected. The authors found that a set of neurons and endocrine cells in *Drosophila* that express a neuropeptide called Allatostatin A adapts the flies to an energy-saving state, by promoting sleep and reducing appetite. They also showed that this cross-talk between sleep and feeding-drive is modulated by the circadian clock.

Animals should also be able to sense their internal reserves of nutrients to make choices regarding when and how much to eat. A study published in *PLOS Biology* [4] discovered how cells in the *Drosophila* fat body (an organ that does a job roughly equivalent to the vertebrate liver and adipose tissue) communicate with the brain to inform it about the quantity of energy reserves, and how this information allows the animal to adjust its feeding behavior accordingly. The authors found that fat body cells contain two of the three enzymes needed to make a molecule called tetrahydrobiopterin (BH4); the third is found in a small subset of neurons in the brain. Reducing the activity of any of these enzymes increased the flies’ appetite, an effect that could be counteracted by including BH4 in their food. The authors were able to show that BH4 acts as a fat-derived signal that induces satiety by inhibiting the propensity of those neurons to secrete a neuropeptide homologous to NPY, known to increase appetite in vertebrates.

A separate study in *PLOS ONE* [5] identified a different set of neurons that also promote feeding; these neurons express a receptor for the inhibitory neurotransmitter GABA. When the authors knocked down the expression of this GABA receptor, the flies over-consumed different tastants. In addition, acute activation of these neurons was sufficient to drive consumption of appetitive and non-appetitive substances, while acute silencing of these neurons had the opposite effect.

In addition to controlling protein intake, animals must finely regulate the amount of sugar they consume. The authors of a recently published study in *eLife* [6] found that a sensory neuron that expresses the receptor IR60b limits sucrose intake in *Drosophila*. The discovery of this receptor and the neuron that expresses it provides the molecular and cellular elements of a new circuit that regulates feeding. Controlling feeding bouts is a common strategy among animals to regulate their food intake; *C*. *elegans*, for example, eats by pumping bacteria from the environment to the gut, and research published in *Nature Communications* [7] showed that this animal fine-tunes its feeding response to the concentration of nutritious bacteria present in the environment by modulating the bouts of fast pumping. By carefully analyzing the feeding behavior of several *C*. *elegans* mutants, the authors concluded that serotonin promotes bouts of pumping in response to food availability via its receptors SER-1 and SER-4.

A study published recently in *PLOS ONE* [8] investigated how the expression of gustatory receptors in the honeybee *Apis mellifera* changes with age and feeding-status, reflecting both the satiety state as well as the type of sugar they have fed. The elegant mechanism that coordinates changes in expression of chemosensory receptors in response to feeding-state was the subject of a recent paper published in *PLOS Genetics* [9]. Every one of the 302 neurons in *C*. *elegans* has been identified, and it is known that the sensory neuron ADL expresses the chemosensory receptor srh-234. The authors found that the expression of this receptor is regulated both cell-autonomously (by a set of transcription factors in neuron ADL) and by non-cell-autonomous mechanisms involving distinct transcription factors in the intestine (but dependent on insulin signaling in the sensory neuron).

For more detailed reading please see the associated PLOS Collection [10].
